# PBMDA: A novel and effective path-based computational model for miRNA-disease association prediction

**DOI:** 10.1371/journal.pcbi.1005455

**Published:** 2017-03-24

**Authors:** Zhu-Hong You, Zhi-An Huang, Zexuan Zhu, Gui-Ying Yan, Zheng-Wei Li, Zhenkun Wen, Xing Chen

**Affiliations:** 1 Xinjiang Technical Institute of Physics and Chemistry, Chinese Academy of Science, ürümqi, China; 2 College of Computer Science and Software Engineering, Shenzhen University, Shenzhen, China; 3 Academy of Mathematics and Systems Science, Chinese Academy of Sciences, Beijing, China; 4 School of Computer Science and Technology, China University of Mining and Technology, Xuzhou, China; 5 School of Information and Control Engineering, China University of Mining and Technology, Xuzhou, China; University of Calgary Cumming School of Medicine, CANADA

## Abstract

In the recent few years, an increasing number of studies have shown that microRNAs (miRNAs) play critical roles in many fundamental and important biological processes. As one of pathogenetic factors, the molecular mechanisms underlying human complex diseases still have not been completely understood from the perspective of miRNA. Predicting potential miRNA-disease associations makes important contributions to understanding the pathogenesis of diseases, developing new drugs, and formulating individualized diagnosis and treatment for diverse human complex diseases. Instead of only depending on expensive and time-consuming biological experiments, computational prediction models are effective by predicting potential miRNA-disease associations, prioritizing candidate miRNAs for the investigated diseases, and selecting those miRNAs with higher association probabilities for further experimental validation. In this study, Path-Based MiRNA-Disease Association (PBMDA) prediction model was proposed by integrating known human miRNA-disease associations, miRNA functional similarity, disease semantic similarity, and Gaussian interaction profile kernel similarity for miRNAs and diseases. This model constructed a heterogeneous graph consisting of three interlinked sub-graphs and further adopted depth-first search algorithm to infer potential miRNA-disease associations. As a result, PBMDA achieved reliable performance in the frameworks of both local and global LOOCV (AUCs of 0.8341 and 0.9169, respectively) and 5-fold cross validation (average AUC of 0.9172). In the cases studies of three important human diseases, 88% (Esophageal Neoplasms), 88% (Kidney Neoplasms) and 90% (Colon Neoplasms) of top-50 predicted miRNAs have been manually confirmed by previous experimental reports from literatures. Through the comparison performance between PBMDA and other previous models in case studies, the reliable performance also demonstrates that PBMDA could serve as a powerful computational tool to accelerate the identification of disease-miRNA associations.

## Author summary

Identification of miRNA-disease associations is considered as a key way for the development of pathology, diagnose and therapy. Computational prediction models contribute to discovering the underlying disease-related miRNAs on a large scale. Based on the assumption that functionally related miRNAs tend to be involved in phenotypically similar disease and vice versa, the model of PBMDA was developed to prioritize the underlying miRNA-disease associations by adopting a special depth-first search algorithm in a heterogeneous graph, which was composed of known miRNA-disease association network, miRNA similarity network, and disease similarity network. Through leave-one-out cross validation and 5-fold cross validation, the promising results demonstrated the effectiveness of the proposed model. We further implemented the case studies of three important human complex diseases, 88%, 88% and 90% of top-50 predicted miRNA-disease associations have been manually confirmed based on recent experimental reports. It is anticipated that PBMDA could prioritize the most potential miRNA-disease associations on a large scale for advancing the progress of biological experiment validation in the future, which could further contribute to the understanding of complex disease mechanisms.

## Introduction

MicroRNAs (miRNAs) are an abundant class of small (20~25 nucleotides) endogenous non-coding RNAs, which were normally deemed as negative gene regulators by suppressing the expression of messenger RNAs (mRNAs) in a sequence-specific manner and repressing the protein translation of their target genes [1–4]. Since the two members of the miRNA family (i.e., *lin-4* and *let-7*) were firstly discovered [5–7], mounting biological observations and studies have indicated that miRNAs play important roles in many important biological processes. To date, 2588 miRNAs have been discovered in the human genome [8]. With the advances in molecular biology and biotechnology, miRNAs have been proven to influence many important physiological processes such as cell growth [9], immune reaction [10], cell differentiation [11], cell development [9], cell cycle regulation [12], inflammation [13], cell apoptosis [14], stress response [9,15], and tumor invasion [16]. In addition, emerging evidences imply the strong links between miRNAs and diseases. For examples, *miR-129*, *miR-142-5p*, and *miR-25* were found to be differentially expressed in all pediatric brain tumor types [17]. *MiR-145* was observed to target the insulin receptor substrate-1 and restrain the growth of colon cancer cells [18]. *MiR-23/27/24* clusters were involved in angiogenesis and endothelial apoptosis, which has potential therapeutic applications in both of vascular disorders and ischemic heart disease [19]. Therefore, the accumulating miRNA-disease associations could be utilized for the pathological classification, individualized diagnosis, and disease treatment [20].

Some databases (e.g. HMDD [21] and miR2Disease [22]) have been constructed for providing accumulating insights into the relationship between miRNAs and diseases. To date, HMDD has already collected 10368 entries including 572 miRNAs and 378 diseases, which shed some light on the molecular mechanisms of diseases. The existing knowledge of miRNA-disease associations mainly comes from previous biological experiments. However, only depending on the traditional biological experiments is time-consuming and costly, therefore unpractical to detect miRNA-disease associations on a large scale in a short term. For addressing these challenges, increasing attentions have been devoted to developing computational models to predict potential miRNA-disease associations by integrating various experimentally confirmed and heterogeneous datasets [23–29].

For predicting or prioritizing disease-related miRNAs, there have been some computational methods proposed, which are mostly based on the assumption that functionally related miRNAs tend to be involved in phenotypically similar diseases and vice versa. For examples, Jiang *et al*. [30] constructed functionally related miRNA network and human phenome-microRNAome network and prioritized the potential miRNA-disease associations according to the cumulative hypergeometric distribution. However, this method excessively depended on the predicted miRNA-target associations which include a high rate of false-positive and high false-negative results. Xuan *et al*. [31] presented the prediction model of HDMP based on the most weighted similar neighbors to predict potential disease-associated miRNAs. This method integrated the information content of disease terms and phenotype similarity between diseases to infer the functional similarity of miRNA pairs. However, it becomes invalid when no known associated miRNA is available for diseases which are less investigated. Mørk *et al*. [32] developed a protein-driven method called miRPD for inferring miRNA-disease associations by calculating the product of the two scoring functions, which were calculated based on the miRNA-protein and protein-disease associations. These three aforementioned methods only considered miRNA neighbor information in their ranking system, which can be summarized as traditional local network similarity measure-based computational models. Some methods were mainly proposed based on the more effective global network similarity measure rather than traditional local network similarity measures. For example, Chen *et al*. [33] proposed the computational model of Random Walk with Restart for MiRNA-Disease Association (RWRMDA) to identify novel disease-related miRNAs by implementing random walks on the miRNA-miRNA functional similarity network. However, this model cannot work for new diseases without any known related miRNAs. Similarly, Xuan *et*.*al*. [34] developed a computational model of MIRNAs associated with Diseases Prediction (MIDP) and its extension version named MIDPE for the diseases with known related miRNAs and without any known related miRNAs, respectively. They established the transition matrices between the labeled and unlabeled nodes for exploring the prior information of nodes and the different ranges of topologies. Shi *et*.*al*. [35] also performed random walk analysis to explore the relationships between miRNAs and diseases by calculating the functional associations between miRNA targets and disease genes in protein-protein interaction networks. Later, Chen *et al*. [36] developed the method of Within and Between Score for MiRNA-Disease Association prediction (WBSMDA) to uncover the potential miRNA-disease associations by integrating several heterogeneous biological datasets, which improves the prediction accuracy of previous classical computational models. By calculating and combining Within-Score and Between-Score, WBSMDA can work for new diseases without any known related miRNAs and new miRNAs without associated diseases. Some proposed machine learning-based models demonstrated their power in this field. Based on the assumption that aberrant regulations of target mRNAs occur because their miRNAs are implicated in a specific disease, Xu *et al*. [37] prioritized novel disease-related miRNAs by constructing the MiRNA Target-Dysregulated Network (MTDN). In addition, they built a support vector machine (SVM) based supervised classifier to distinguish prostate cancer and non-prostate cancer related miRNAs by combining four topological features extracted from MTDN. However, the prediction performance suffered from unavailable verified negative miRNA-disease association samples. A semi-supervised method called Regularized Least Squares for MiRNA-Disease Association (RLSMDA) was developed by Chen *et al*. [38]. Considering obtaining verified negative samples (i.e. those disease-miRNA pairs without any known association evidences) is difficult and even impossible, cost functions were defined and minimized in the framework of Regularized Least Squares (RLS). Then the optimal classifiers in the disease and miRNA spaces were yielded and combined to obtain the final predictive results. Chen *et al*. [39] also developed the model of Restricted Boltzmann Machine (RBM) for multiple types of miRNA-disease association prediction (RBMMMDA) for predicting various types of miRNA-disease associations. Based on the known miRNA-disease association network, RBMs were constructed and trained by Contrastive Divergence (CD) algorithm. Finally, this model implemented prediction by calculating conditional probabilities.

The knowledge on miRNAs provides valuable information for the prevention, diagnosis and treatment of human diseases. There is an urgent need to accelerate the identification of disease-miRNA associations for further studies on pathology as well as drug development. We here proposed a novel Path-Based MiRNA-Disease Association (PBMDA) prediction method by constructing a heterogeneous graph consisting of three interlinked sub-graphs (i.e., miRNA-miRNA similarity network, disease-disease similarity network and known miRNA-disease association network). Integrating different types of heterogeneous biological datasets allows that PBMDA could be applied to the new diseases with no known associated miRNAs and the new miRNAs with no known associated diseases. In addition, the proposed method can simultaneously prioritize all unknown miRNAs for all the investigated diseases.

In this work, three evaluation frameworks, including global leave-one-out cross validation (global LOOCV), local leave-one-out cross validation (local LOOCV) and 5-fold cross validation (5-fold CV), were implemented to evaluate the prediction performance of PBMDA. When performing on the HMDD database, PBMDA obtained the best performance in the frameworks of both local and global LOOCV (AUCs of 0.8341 and 0.9169, respectively) and 5-fold cross validation (average AUC of 0.9172) compared with several state-of-the-art computational models [31,33,36]. To further evaluate the performance of PBMDA, we implemented case studies of three important human diseases. As a result, most of top-50 predicted disease-related miRNAs (44/50 for *Esophageal Neoplasms*; 44/50 for *Kidney Neoplasms*; 45/50 for *Colon Neoplasms*) were verified by previously published literatures, respectively. Besides, 9 out of top-10 predicted obesity-related were manually validated based on the published literatures. Our model also represents an improvement to the prediction accuracy through the comparison performance between PBMDA and other previous representative models in case studies. A simulation experiment also proves the applicability of our model to a new disease (no known associated miRNAs).

## Materials and methods

### Human miRNA-disease associations

HMDD database (http://www.cuilab.cn/hmdd) has collected 5430 experimentally verified human miRNA-disease associations (see S1 Table), involving 495 miRNAs and 383 diseases (see S2 and S3 Tables). The adjacency matrix *Y* is constructed to describe the confirmed associations between miRNA and disease. Namely, if miRNA *m*(*i*) is recorded to be associated with disease *d*(*j*), the entity *Y*(*i*,*j*) is equal to 1, otherwise 0. For further description in detail, the investigated numbers of miRNAs and diseases in our study are represented by variables *nm* and *nd*, respectively. To evaluate the prediction lists for case studies, another two independent databases (i.e. dbDEMC [40] and miR2Disease [22]) are utilized for validation.

### MiRNA functional similarity

According to previous literature [41], it could be concluded that miRNAs with similar functions are more likely associated with similar diseases. Under this assumption, miRNA functional similarity score was calculated (http://www.cuilab.cn/files/images/cuilab/misim.zip). We therefore utilized these data to construct miRNA functional similarity symmetric matrix *FS* (see S4 Table), in which the entity *FS*(*m*(*i*),*m*(*j*)) indicates how is miRNA *m*(*i*) functionally similar to another miRNA *m*(*j*).

### Disease semantic similarity

Mesh database (http://www.ncbi.nlm.nih.gov/), a strict system for disease classification, is available for effectively researching the relationship between different diseases. Disease could be transformed into corresponding Directed Acyclic Graph (DAG), such as *DAG*(*D*) = (*T*(*D*),*E*(*D*)), where *T(D)* indicates the node set including node *D* and its ancestor nodes, and *E*(*D*) is the edge set of corresponding direct links from a parent node to a child node, which represents the relationship between different diseases [41]. Based on disease DAG, the contribution of disease term *d* to the semantic value of disease *D* and the semantic value of disease *D* itself can be formulated by the following two equations, respectively.

$$\left\{ \begin{array}{l} D_{D}(d)=1\;if\;d=D\\ D_{D}(d)=\max\left\{\Delta_{*}D_{D}(d')|d'\in children\;of\;d\right\}\;if\;d\neq D \end{array}\right.$$ (1)

$$DV(D)=\sum_{d\in T(D)}D_{D}(d)$$ (2)

Where △ is a the semantic contribution decay factor, which shows that as the distances between disease *D* and its ancestor diseases increases, their contribution to the semantic value of disease *D* progressively decreases. Accordingly, disease *D* locates in the 0th layer, the contribution to the semantic value of disease *D* itself was defined as 1. The contribution of its ancestor disease should be multiplied by the semantic contribution decay factor. Therefore, △ should be assigned a value between 0 and 1, and that this value was set as 0.5 here according to some previous important literatures [42,43]. Based on this way to measure disease semantic similarity, it should be considered that two diseases sharing more common parts of their DAGs should obtain higher semantic similarity. Under this assumption, the semantic similarity between two diseases *d*(*i*) and *d*(*j*) can be calculated as:

$$SS\left(d\left(i\right),d\left(j\right)\right)=\frac{\sum_{t\in T\left(d\left(i\right)\right)\cap T\left(d\left(j\right)\right)}\left(D_{d\left(i\right)}\left(t\right)+D_{d\left(j\right)}\left(t\right)\right)}{DV\left(d\left(i\right)\right)+DV\left(d\left(j\right)\right)}$$ (3)

where the entity *SS*(*d*(*i*),*d*(*j*)) in row *i* column *j* represents the disease semantic similarity between *d*(*i*) and *d*(*j*).

### Gaussian interaction profile kernel similarity for diseases

According to the basic assumption that two miRNAs with more functional similarity tend to be more associated with similar diseases, the topologic information of the known miRNA-disease association network could be used to measure disease similarity. We therefore introduce Gaussian interaction profile kernel for calculating the network topologic similarity between diseases. A binary vector *IP*(*d*(*i*)), i.e. the *ith* column of matrix *Y*, is recorded as the interaction profiles of disease *d*(*i*) for representing associations between *d*(*i*) itself and each miRNA. We then utilized Eq (4) to compute Gaussian kernel similarity between disease *d*(*i*) and disease *d*(*j*) based on their interaction profiles.

$$KD\left(d\left(i\right),d\left(j\right)\right)=\exp\left(-\gamma_{d}{\left\|IP\left(d\left(i\right)\right)-IP\left(d\left(j\right)\right)\right\|}^{2}\right)$$ (4)

where parameter *γ*_*d*_ is a regulation parameter of the kernel bandwidth. As a symmetric matrix, *KD* represents the Gaussian interaction profile kernel similarity for all investigated diseases. Parameter *γ*_*d*_ is needed to be updated by using a new bandwidth parameter *γ*^'^_*d*_ divided by the average value of associations with miRNAs for all diseases. Based on the previous successful research about lncRNA-disease association prediction [44], *γ*^'^_*d*_ is set to 1 for controlling the kernel bandwidth. So *γ*_*d*_ can be formulated as:

$$\gamma_{d}=\gamma^{'}{}_{d}/\left(\frac{1}{nd}\sum_{i=1}^{nd}{\left\|IP\left(d\left(i\right)\right)\right\|}^{2}\right)$$ (5)

### Gaussian interaction profile kernel similarity for miRNAs

Similarly, we also calculated the Gaussian interaction profile kernel similarity for miRNAs, which can be calculated by Eqs (6) and (7):

$$KM\left(m\left(i\right),m\left(j\right)\right)=\exp\left(-\gamma_{m}{\left\|IP\left(m\left(i\right)\right)-IP\left(m\left(j\right)\right)\right\|}^{2}\right)$$ (6)

$$\gamma_{m}=\gamma^{'}{}_{m}/\left(\frac{1}{nm}\sum_{i=1}^{nm}{\left\|IP\left(m\left(i\right)\right)\right\|}^{2}\right)$$ (7)

where *γ*^'^_*m*_ = 1 and *KM* is a symmetric matrix, whose entity *KM*(*m*(*i*),*m*(*j*)) denotes the Gaussian interaction profile kernel similarity between miRNA *m*(*i*) and miRNA *m*(*j*).

### Integrated similarity for miRNA and disease

MiRNA functional similarity and disease semantic similarity are the primary data to construct the disease and miRNA similarity matrix. However, these matrices have the problem of sparsity, so we calculated the Gaussian interaction profile kernel similarity based on the known miRNA-disease associations for calculating the similarity of those disease-disease or miRNA-miRNA pairs without corresponding disease semantic similarity or miRNA functional similarity. For constructing two integrated similarity matrix (i.e., miRNA similarity matrix *S*_*m*_ and disease similarity matrix *S*_*d*_), we integrated miRNA functional similarity, disease semantic similarity, and Gaussian interaction profile kernel similarity for miRNAs and diseases by judging whether miRNA *m*(*i*)/disease *d*(*i*) has functional/semantic similarity with another miRNA *m*(*j*)/disease *d*(*j*) or not.

$$S_{m}\left(m\left(i\right),m\left(j\right)\right)=\left\{ \begin{array}{l} FS\left(m\left(i\right),m\left(j\right)\right)\;m\left(i\right)\;and\;\text{m}\left(j\right)\;has\;functional\;similarity\\ KM\left(m\left(i\right),m\left(j\right)\right)\;otherwise \end{array}\right.$$ (8)

$$S_{d}\left(d\left(i\right),d\left(j\right)\right)=\left\{ \begin{array}{l} SS\left(d\left(i\right),d\left(j\right)\right)\;d\left(i\right)\;and\;d\left(j\right)\;has\;semantic\;similarity\\ KD\left(d\left(i\right),d\left(j\right)\right)\;otherwise \end{array}\right.$$ (9)

### PBMDA

**Fig 1:**
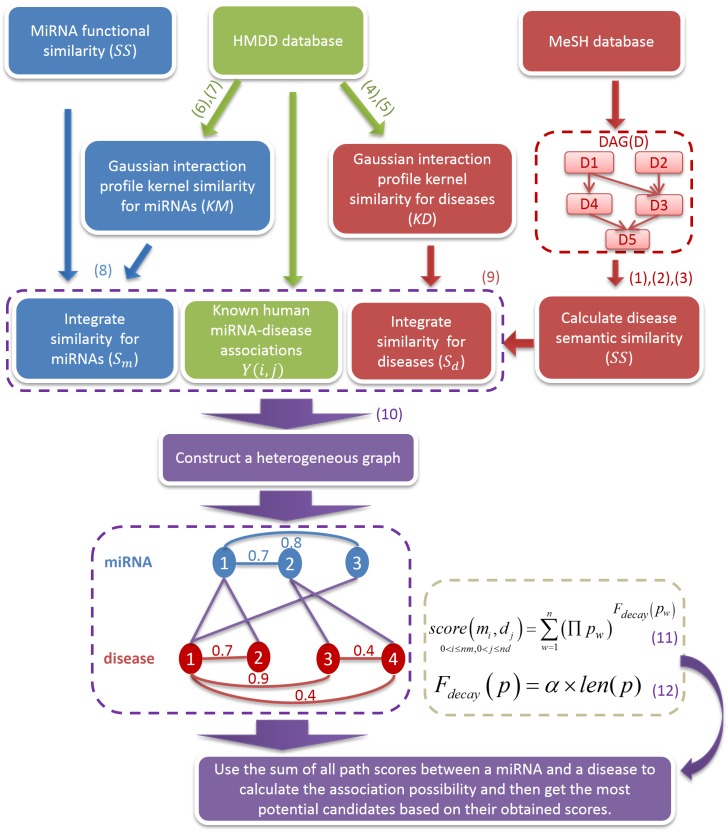
Flowchart of PBMDA. Three networks were integrated to construct a heterogeneous graph and the potential miRNA-disease associations could be effectively inferred by the scoring system.

For prioritizing the most possible potential miRNA-disease associations, we here devised a novel Path-Based MiRNA-Disease Association (PBMDA) prediction method (See Fig 1). For eliminating the node sets with the weak interaction, we set the threshold variable ***T*** to 0.5 based on the previous literature research [45], which means we did not take such links into consideration if the similarity between these nodes was less than 0.5. So that three weighted matrixes can be represented as:

$$\begin{array}{l} W_{miRNA-miRNA}=\left\{ \begin{array}{l} 0\;S_{m}\left(m\left(i\right),m\left(j\right)\right)<T\\ S_{m}\left(m\left(i\right),m\left(j\right)\right)\;otherwise \end{array}\right.\\ W_{disease-disease}=\left\{ \begin{array}{l} 0\;S_{d}\left(d\left(i\right),d\left(j\right)\right)<T\\ S_{d}\left(d\left(i\right),d\left(j\right)\right)\;otherwise \end{array}\right.\\ W_{miRNA-disease}=Y\left(m_{i},d_{j}\right)\;0\leq i\leq nm,0\leq j\leq nd \end{array}$$ (10)

In this way, we constructed a heterogeneous graph with lots of paths, which consisted of these three weighted matrixes. A path was defined as a connection between a miRNA and a disease. Furthermore, all paths between a miRNA and a disease must be acyclic to avoid the visited nodes from being traversed repeatedly. A specific depth-first search algorithm was adopted to traverse all paths in the graph, which is easy to be implemented as a recursive algorithm. For saving time, we set a parameter ***L*** to limit the maximum length of paths. According to previous literature research [45] and comparison experiments, we found that it was suitable to set ***L*** = 3 after trying different values from 2 to 4 increasingly, i.e. one path was not allowed to include more than three edges. Based on the assumption that if more paths are found to connect a miRNA and a disease, they are more likely to have associations, the accumulative contributions from all paths between a miRNA-disease pair could be integrated as a final score. Accordingly, the scoring formula can be defined as Eq (11) with the exponential decay function *F*_*decay*_(*p*), which is depended on the path between a specific miRNA *m*_*i*_ and a specific disease d_j_:

$$\underset{0<i\leq nm,0<j\leq nd}{score\left(m_{i},d_{j}\right)}={\sum_{w=1}^{n}\left(\prod p_{w}\right)}^{F_{decay}\left(p_{w}\right)}$$ (11)

where *p* = {*p*_1_,*p*_2_,…,*p*_*n*_} is a set of paths linking up a miRNA *m*_*i*_ and a disease d_j_, and ∏*p*_*w*_ represents the product of the weight of the all the edges in path *p*_*w*_ obtained from the Eq (10).

Generally, longer paths between a miRNA and a disease should have less confidence to directly demonstrate their relationship, i.e. the contributions from the longer path should be cut down more sharply. So the decay function *F*_*decay*_(*p*) can be calculated as follows:

$$F_{decay}\left(p\right)=\alpha \times len(p)$$ (12)

where parameter *α* is a decay factor, which was set 2.26, according to previous literature research [45], and *len*(*p*) is the length of path *p*. After traversing all paths in the graph, each miRNA-disease pair could obtain a final score representing the association confidence between this miRNA and disease, i.e. the higher score they obtain, the more closely related they should be. As an example in Fig 1, the score value of *miRNA*1 (*m*_1_) and *disease*1 (*d*_1_) is calculated as: 1.0^2.26*1^ < *m*_1_ ↔ *d*_1_ > + (1.0 * 0.7)^2.26*2^ < *m*_1_ ↔ *d*_2_ ↔ *d*_1_ > + (0.8 * 1.0)^2.26*2^ < *m*_1_ ↔ *m*_3_ ↔ *d*_1_ > + (0.7 * 1.0 * 0.9)^2.26*3^ < *m*_1_ ↔ *m*_2_ ↔ *d*_3_ ↔ *d*_1_ > ≈ 1.6078. The edge between *d*_4_ and *d*_1_ is not taken into consideration, because its weight is less than threshold ***T***. The code and data of PBMDA is freely available at http://www.escience.cn/system/file?fileId=84394.

### LOOCV and 5-fold CV

To evaluate the predictive performance of PBMDA, we implemented LOOCV and 5-fold CV based on known miRNA-disease associations downloaded from HMDD database [21]. LOOCV could be divided into two evaluation frameworks based on the ranking scope (i.e., global LOOCV considers all investigated diseases while local LOOCV only includes a given disease). They both followed the common framework of LOOCV, i.e. each known miRNA-disease association was left in turns served as a test sample and other known miRNA-disease associations were regarded as training samples. Test sample was ranked among the candidate miRNA-disease associations without any known association evidences. The test samples with higher ranks than the specific threshold would be considered as successful predictions. In the framework of 5-fold CV, all known verified miRNA-disease associations were randomly divided into five uncrossed groups, of which one was regarded as testing samples and the other four were used for training in turns. In this paper, we randomly implemented 100 divisions of all known verified miRNA-disease associations to reduce bias brought by sample divisions. The receiver operating characteristic (ROC) curves were drawn for performance evaluation by calculating true positive rate (TPR, sensitivity) and false positive rate (FPR, 1-specificity) based on the varying threshold. Sensitivity indicates the percentage of the positive test samples which are ranked higher than the given threshold; specificity indicates the percentage of candidate samples which are ranked lower than the given threshold. In this way, the ROC curves were plotted based on TPR versus FPR. The areas under ROC curves (AUCs) were also calculated for a numerical evaluation of model performance. AUC = 0.5 denotes a purely random prediction while AUC = 1 denotes a perfect prediction. As a result, the reliable AUCs of 0.9169 and 0.8341 in the frameworks of global and local LOOCV were obtained by PBMDA. Furthermore, the average and the standard deviation of AUC in the framework of 5-fold CV are 0.9172 and 0.0007, respectively. It is anticipated that PBMDA could serve as an effective and robust computational prediction model.

## Results

### Performance comparison with other methods

**Fig 2:**
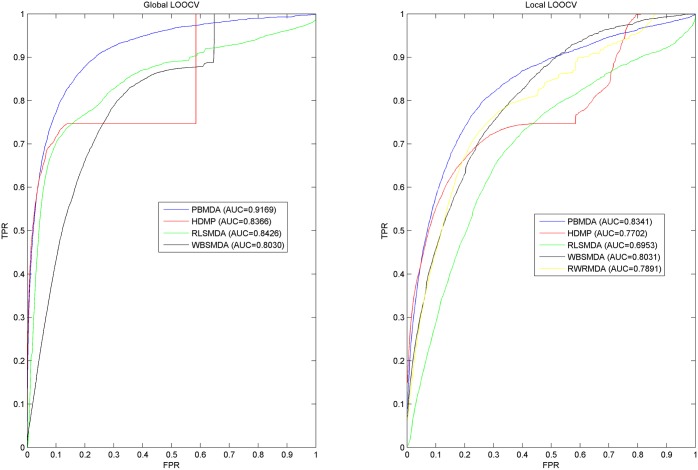
The comparison results between PBMDA and other four computational models in terms of global LOOCV and local LOOCV.

We further compared the prediction performance of PBMDA model with four state-of-the-art computational prediction models (i.e., WBSMDA [36], RLSMDA [38], HDMP [31] and RWRMDA [33]). RWRMDA and HDMP are the representational methods in this domain. They were often chosen as benchmarking methods to validate the later developed methods, such as: MIDP [35] and Shi’s method [36]. RLSMDA was a semi-supervised learning method based on the framework of Regularized Least Squares (RLS) representing a good try in machine learning algorithm. WBSMDA was a newly published method representing the current level of computational prediction models in this domain. The performance comparisons in the framework of global and local LOOCV were shown in Fig 2. As a result, PBMDA, WBSMDA, RLSMDA and HDMP achieved AUCs of 0.9169, 0.8030, 0.8426 and 0.8366 in the framework of global LOOCV, respectively. When implementing the local LOOCV, we also obtain the best prediction performance based on PBMDA with AUC of 0.8341. The other methods (WBSMDA, RWRMDA, HDMP, and RLSMDA) obtained AUCs of 0.8031, 0.7891, 0.7702 and 0.6953, respectively. In addition, 5-fold CV was implemented on PBMDA, WBSMDA, RLSMDA and HDMP with average AUC value of 0.9172+/-0.0007, 0.8185+/-0.0009, 0.8569+/-0.0020 and 0.8342+/-0.0010, respectively, which was observed that PBMDA obtained the best performance based on 5-fold CV. In conclusion, PBMDA significantly improves prediction performance of previous computational models by demonstrating its reliable and robust performance from these evaluation frameworks.

### Effects of parameters

For simplicity, the maximum path length ***L*** and weight threshold ***T*** were respectively selected 3 and 0.5 for the prediction. It is possible to obtain the better prediction performance by adjusting these parameters values. 5-fold CV was implemented over 100 times for further evaluation. As a result, given ***T*** = 0.5, the average AUC respectively equals to 0.7726+/-0.0016 (when ***L*** = 2) and 0.9172+/-0.0007 (when ***L*** = 3). It seemed that, the performance of our model could be affected by being restrained with the insufficient neighbor length in each path. Because it took too long to run the program, we did not complete the experiment when ***L*** = 4. It seems that as ***L*** increases, the computational complexity represents an exponential growth. However, we also implemented 5-fold CV 100 times based on our model and a smaller dataset (HMDD v1.0, 1395 known miRNA-disease associations involved in 271 miRNAs and 137 diseases) for testing performance with increasing ***L*** (***L*** = 2~4) and fixed ***T*** (***T*** = 0.5). As we can see in Table 1, the prediction accuracy of proposed model is deceased with the increasing ***L*** based on a smaller dataset. It is assumed that the increasing ***L*** tends to cause overfitting problems for a small network dataset. Therefore, it is not necessary to obtain the improvement of prediction accuracy by increasing the path length ***L***. We should take the size of a network dataset into consideration when selecting parameter ***L***. It seems like that ***L*** should be generally increased, as the network dataset becomes larger. Nevertheless, it does not need to select a too high numerical value for ***L*** for avoiding overfitting. Considering the reliable performance of three maximum limited lengths for each path (i.e., ***L*** = 3), there is no necessity to select ***L*** = 4 in this study. This setting also helps reduce the run-time of computational model to travel all possible paths.

**Table 1:** Five-fold CV experiment results of changing a parameter *L* when *T* = 0.5 based on a smaller dataset (HMDD v1.0).

*L*	2	3	4
Average AUC	0.9175+/-0.0022	0.8823+/-0.0021	0.8443+/-0.0019

We also implemented a series of 5-fold CV experiments with the increasing ***T*** values to obtain the optimal setting (see Table 2). It is showed that, the selection of ***T*** value cannot greatly affect the accuracy of PBMDA, which demonstrates a strong robustness on this parameter. So we made a better trade-off to set ***T*** = 0.5.

**Table 2:** Five-fold CV experiment results of changing a parameter *T* when *L* = 3.

*T* value	Average AUCs	Standard deviation
0.2	0.9174	0.0007
0.3	0.9174	0.0007
0.4	0.9173	0.0007
0.5	0.9172	0.0007
0.6	0.9168	0.0007
0.7	0.9158	0.0007
0.8	0.9138	0.0009

### Case studies

Many miRNAs in the top rank were predicted to have associations with digestive system and urinary system. It seems that miRNA functional expression is closely related to the dysfunction of both digestive system and urinary system. We attempted to explore their potential relationships and what a role of miRNA plays in disease mechanisms of digestive and urinary system. Esophagus and colon belong to digestive system, while kidney belongs to urinary system. Therefore, to further evaluate the prediction performance of PBMDA, *Esophageal Neoplasms*, *Kidney Neoplasms* and *Colon Neoplasms* were investigated to infer their underlying associated miRNAs. Two independent databases (i.e., dbDEMC [40] and miR2Disease [22]) were used as benchmark datasets to verify the predictive results. The quantitative statistics demonstrates the reasonability of this benchmarking method (see Table 3). Besides, miR2Disease and dbDEMC are commonly utilized to be benchmark datasets in this domain, such as HDMP and MIDP models. It is worthwhile to note that, the predicted miRNA-disease associations were not included in HMDD database, including those highly-ranked disease-related miRNAs listed in Tables 4–7. For the sake of space, this article only mentions these three cancers. Actually, our model can make a successful prediction for almost all of given important diseases. We also present the verification of top-50 prediction list for those important diseases investigated by other previous computational models, such as: prostate neoplasms, breast neoplasms and lung neoplasms in HDMP (see Table 8 and S5 Table). Although HMDD database included plentiful of known miRNA-disease associations, there were still many other miRNA-disease associations existing in other two independent benchmark databases, which were not overlapped in HMDD database. So that, we utilized the known miRNA-disease associations (included in HMDD) to prioritize the novel miRNA-disease associations (not included in HMDD), and evaluated the prediction performance of PBMDA by observing how many these novel associations were matched by other two independent benchmark databases.

**Table 3:** The quantitative statistics between test dataset (HMDD) and benchmark datasets (miR2Disease and dbDEMC).

Database	Total associations without repetition *abbr*. A	Redundant associations removed from HMDD *abbr*. B	Remainder associations (A-B)	Percentage of remainder associations $\frac{A-B}{5430}*100\%$
HMDD	5430	/	/	/
miR2Disease	2875	232	2643	48.7%
dbDEMC	1815	546	1269	23.4%

**Table 4:** PBMDA was applied to Esophageal Neoplasms to predict the potential disease-related miRNAs, and 44 of top-50 predicted miRNAs have been confirmed according to recent experimental literatures.

Top 1–25		Top 26–50	
miRNA	Evidence	miRNA	Evidence
hsa-mir-17	dbdemc	hsa-mir-195	dbdemc
hsa-mir-125b	dbdemc	hsa-let-7g	dbdemc
hsa-mir-221	dbdemc	hsa-mir-124	dbdemc
hsa-mir-16	dbdemc	hsa-let-7i	dbdemc
hsa-mir-18a	dbdemc	hsa-mir-125a	dbdemc
hsa-mir-200b	dbdemc	hsa-mir-24	dbdemc
hsa-mir-19b	dbdemc	hsa-mir-106b	dbdemc
hsa-mir-1	dbdemc	hsa-mir-93	dbdemc
hsa-mir-218	unconfirmed	hsa-mir-30c	dbdemc
hsa-mir-222	dbdemc	hsa-mir-199b	dbdemc
hsa-mir-182	dbdemc	hsa-mir-224	dbdemc
hsa-let-7d	dbdemc	hsa-mir-106a	dbdemc
hsa-mir-29a	dbdemc	hsa-mir-107	dbdemc;miR2Disease
hsa-mir-181a	dbdemc	hsa-mir-127	dbdemc
hsa-mir-29b	dbdemc	hsa-mir-429	dbdemc
hsa-mir-146b	dbdemc	hsa-mir-27b	dbdemc
hsa-mir-10b	dbdemc	hsa-mir-103a	unconfirmed
hsa-mir-181b	dbdemc	hsa-mir-96	dbdemc
hsa-mir-133b	dbdemc	hsa-mir-18b	dbdemc
hsa-mir-9	dbdemc	hsa-mir-151a	unconfirmed
hsa-let-7e	dbdemc	hsa-mir-122	unconfirmed
hsa-mir-142	dbdemc	hsa-mir-135a	dbdemc
hsa-mir-30a	dbdemc	hsa-mir-302b	dbdemc
hsa-let-7f	unconfirmed	hsa-mir-335	dbdemc
hsa-mir-7	dbdemc	hsa-mir-138	unconfirmed

**Table 5:** PBMDA was applied to Kidney Neoplasms to predict the potential disease-related miRNAs, and 44 of top-50 predicted miRNAs have been confirmed according to recent experimental literatures.

Top 1–25		Top 26–50	
miRNA	Evidence	miRNA	Evidence
hsa-mir-155	dbdemc	hsa-mir-205	unconfirmed
hsa-mir-145	dbdemc	hsa-mir-19b	dbdemc;miR2Disease
hsa-mir-146a	dbdemc	hsa-mir-181a	dbdemc
hsa-mir-126	dbdemc;miR2Disease	hsa-mir-218	dbdemc
hsa-mir-125b	unconfirmed	hsa-let-7c	dbdemc
hsa-mir-20a	dbdemc;miR2Disease	hsa-mir-222	dbdemc
hsa-mir-17	dbdemc;miR2Disease	hsa-mir-203	dbdemc
hsa-mir-34a	dbdemc	hsa-mir-9	dbdemc
hsa-mir-16	dbdemc	hsa-mir-34c	dbdemc
hsa-mir-200b	dbdemc;miR2Disease	hsa-mir-10b	dbdemc
hsa-let-7a	dbdemc	hsa-mir-146b	dbdemc
hsa-mir-221	unconfirmed	hsa-mir-182	dbdemc;miR2Disease
hsa-mir-143	dbdemc	hsa-mir-375	dbdemc
hsa-mir-92a	unconfirmed	hsa-let-7d	dbdemc
hsa-mir-200a	dbdemc	hsa-mir-27a	dbdemc;miR2Disease
hsa-mir-31	dbdemc	hsa-mir-34b	dbdemc
hsa-mir-18a	dbdemc	hsa-mir-101	dbdemc;miR2Disease
hsa-mir-19a	dbdemc;miR2Disease	hsa-mir-181b	dbdemc
hsa-mir-223	dbdemc	hsa-mir-106b	dbdemc;miR2Disease
hsa-mir-29a	dbdemc;miR2Disease	hsa-mir-142	unconfirmed
hsa-mir-29b	dbdemc;miR2Disease	hsa-mir-29c	dbdemc;miR2Disease
hsa-mir-1	dbdemc	hsa-mir-195	dbdemc
hsa-let-7b	unconfirmed	hsa-mir-183	dbdemc
hsa-mir-210	dbdemc;miR2Disease	hsa-mir-486	dbdemc
hsa-mir-199a	dbdemc;miR2Disease	hsa-mir-196a	dbdemc

**Table 6:** PBMDA was applied to Colon Neoplasms to predict the potential disease-related miRNAs, and 45 of top-50 predicted miRNAs have been confirmed according to recent experimental literatures.

Top 1–25		Top 26–50	
miRNA	Evidence	miRNA	Evidence
hsa-mir-21	dbdemc;miR2Disease	hsa-mir-181a	dbdemc;miR2Disease
hsa-mir-20a	dbdemc;miR2Disease	hsa-mir-223	dbdemc;miR2Disease
hsa-mir-155	dbdemc;miR2Disease	hsa-let-7c	dbdemc
hsa-mir-146a	dbdemc	hsa-mir-199a	unconfirmed
hsa-mir-18a	dbdemc;miR2Disease	hsa-mir-34c	miR2Disease
hsa-mir-34a	dbdemc;miR2Disease	hsa-mir-9	dbdemc;miR2Disease
hsa-mir-143	dbdemc;miR2Disease	hsa-let-7d	dbdemc
hsa-mir-125b	dbdemc	hsa-mir-15a	dbdemc
hsa-mir-92a	unconfirmed	hsa-mir-10b	dbdemc;miR2Disease
hsa-mir-19b	dbdemc;miR2Disease	hsa-mir-181b	dbdemc;miR2Disease
hsa-mir-16	dbdemc	hsa-mir-106b	dbdemc;miR2Disease
hsa-mir-221	dbdemc;miR2Disease	hsa-mir-34b	dbdemc;miR2Disease
hsa-mir-19a	dbdemc;miR2Disease	hsa-mir-205	dbdemc
hsa-let-7a	dbdemc;miR2Disease	hsa-mir-200a	unconfirmed
hsa-mir-200c	dbdemc;miR2Disease	hsa-mir-203	dbdemc;miR2Disease
hsa-mir-31	dbdemc;miR2Disease	hsa-mir-27a	miR2Disease
hsa-mir-200b	dbdemc	hsa-mir-30a	miR2Disease
hsa-mir-29b	dbdemc;miR2Disease	hsa-mir-133b	dbdemc;miR2Disease
hsa-mir-182	dbdemc;miR2Disease	hsa-mir-101	unconfirmed
hsa-mir-218	dbdemc	hsa-mir-183	dbdemc;miR2Disease
hsa-mir-222	dbdemc	hsa-let-7e	dbdemc
hsa-let-7b	dbdemc	hsa-mir-196a	dbdemc;miR2Disease
hsa-mir-29a	dbdemc;miR2Disease	hsa-let-7f	dbdemc;miR2Disease
hsa-mir-210	dbdemc	hsa-mir-142	unconfirmed
hsa-mir-1	dbdemc;miR2Disease	hsa-mir-148a	dbdemc

**Table 7:** Nine out of top-10 predicted obesity-related miRNAs have been manually validated by the published literatures.

MiRNA	Prediction score	Evidence(PMID)
hsa-mir-20a	223.20	25014161;19348006
hsa-mir-155	222.22	23991091
hsa-mir-145	211.36	22688341
hsa-mir-34a	210.45	22988100
hsa-mir-146a	202.54	23396142
hsa-mir-125b	201.68	23396142
hsa-mir-92a	194.21	22688341
hsa-mir-126	184.72	24749062
hsa-mir-19b	180.86	26658372
hsa-mir-16	175.44	unconfirmed

**Table 8:** Verification of top-50 prediction list for several important human diseases.

Diseases	The verification of top-50 prediction list
Prostate neoplasms	43
Breast neoplasms	33
Lung neoplasms	32
Colon neoplasms	45
Lymphoma	45
Hepatocellular cancer	17

*Esophageal Neoplasms* is one of the most common digestive carcinomas with poor prognosis. With the growth of the tumor, patients could cause corresponding symptoms, such as difficult or painful swallowing, weight loss and coughing up blood. Cisplatin-based chemotherapy is the main approach for the treatment of *Esophageal Neoplasms* but the chemotherapy response is difficultly detected. Some studies suggested that miRNAs could be considered as effective prognostic biomarkers for *Esophageal Neoplasms*. For examples, *hsa-let-7* can be considered as a prognostic biomarker for measuring the response to chemotherapy. In addition, when a recurrence of disease happens, patients have relatively higher expression of mature *hsa-miR-143* and mature *hsa-miR-145* than normal people. A case study of *Esophageal Neoplasms* was implemented on PBMDA for yielding the most probable related miRNAs (see Table 4). As a result, 9 of top-10 and 44 of top-50 candidates were confirmed to have associations with *Esophageal Neoplasms* based on previous experimental literatures. For examples, the overexpression of *hsa-miR-17* (1st in the prediction list) cluster can accelerate the cellular growth in *Esophageal Neoplasms* [46]. Previous research showed that *hsa-miR-125b* (2nd in the prediction list) can promote cell proliferation in *Esophageal Neoplasms* by influencing the target transcripts: *CYP24*, *ERBB2* and *ERBB3* [47]. Moreover, *hsa-miR-221* (3rd in the prediction list) can be regarded as a useful diagnostic marker for measuring the sensitivity to the treatment of *Esophageal Neoplasms* [48].

*Kidney Neoplasms* is the most rapidly increasing tumor type in incidence rate, especially among black persons. And more than 80 percent of patients are found to have renal-cell carcinoma (RCC). Recent studies found that patients with RCC usually have overexpression of *miR-34a*, which plays a critical role in slowing the growth of RCC. Besides, *MiRs-141/200c* were considered as the most down-regulated miRNAs in RCC by targeting *ZEB2*, which is a type of transcriptional repressor [49,50]. In order to identify potential disease-miRNA associations, we implemented the case study of *Kidney Neoplasms*. The existing experimental literatures have demonstrated 9 of top-10 and 44 of top-50 potential miRNA candidates were correctly associated with this important human disease (see Table 5). For example, *miR-155* (1st in the prediction list), *miR-126* (4th in the prediction list) and *miR-20a* (5th in the prediction list) were identified to be upregulated in clear-cell type human renal cell carcinoma (ccRCC), relative to normal kidney samples [51,52]. Furthermore, it was found that *miR-145* (2nd in the prediction list) and *miR-146a* (3rd in the prediction list) with over expression suppress their target mRNA and protein expression of the STAT-1 pathway in kidney tissues [53].

*Colon Neoplasms* maintains the second leading cause of cancer-related death in the United States [54]. Although chemotherapy has important therapeutic value, surgery is still the only curative way for the treatment of *Colon Neoplasms*. There is an urgent need to find potential biomarkers, which have a strong response to the clinical observations. By using in situ hybridization technique, researchers have confirmed that *miR-21* has high expression levels in colonic carcinoma cells [55]. What’s more, *let-7* functions as a potential growth suppressor in human colon cancer tumors and cell lines [56]. Identifying more miRNAs associated with *Colon Neoplasms* helps accurately evaluate the clinical outcomes. Therefore, we implemented the case study of *Colon Neoplasms* based on PBMDA. In the prediction list, 9 of top-10 and 45 of top-50 predicted miRNAs obtained confirmation of their associations with *Colon Neoplasms* based on recent experimental literatures (see Table 6). For examples, preclinical research showed that the expression of *miR-21* (1st in the prediction list) is related to clinicopathologic features of colorectal cancer [57]. Experimental studies also found that *miR-20a* (2nd in the prediction list) shows significantly higher expression in colon cancer tissues than normal tissues [58]. *MiR-18a* (3rd in the prediction list) is considered as a colon tumor suppressor by targeting on *K-Ras* (mRNA) to influence cell proliferation and anchorage-independent growth [59]. What’s more, *miR-34a* (6th in the prediction list) has important potential to be used as potential diagnostic and prognostic biomarker by using its expression at different stages of *Colon Neoplasms* [60].

Considering that most cancers are characterized by some extent of genetic and genomic modifications, so the fact that cancers are associated with miRNA dysregulation is perhaps an obvious form of validation, which inspired us to know whether PBMDA can achieve the similar effectiveness for another disease type, such as obesity. Because there is only one entry corresponding to the miRNA-obesity association in both HMDD and the two independent benchmark databases (i.e., dbDEMC [40] and miR2Disease [22]), we decided to manually validate the top-10 predicted obesity-related miRNAs based on the published literatures. As we can see the validation results from Table 7, 9 out of top-10 predicted miRNAs have been demonstrated to be associated with obesity, which demonstrated that PBMDA also work effectively for other diseases.

Because only WBSMDA and PBMDA chose the latest version of HMDD for the prediction, we decided to compare the performance between PBMDA and WBSMDA by observing how many top-50 predicted miRNA-disease associations have been confirmed by dbdemc and miR2Disease databases for these three important diseases. Their validation results were compared in Table 9. As we can see from this table, PBMDA perform better than WBSMDA in general.

**Table 9:** Performance comparison between PBMDA and WBSMDA in case studies of three important diseases in top-50 prediction list based on the latest version of HMDD (v2.0).

	Esophageal Neoplasms	Kidney Neoplasms	Colon Neoplasms
PBMDA	44	44	45
WBSMDA	29	39	45

Besides, we have also implemented PBMDA on the older version of HMDD (v1.0) for further comparison between PBMDA and another three compared computational models, i.e. HDMP, RWRMDA and RLSMDA. For a fair comparison, we only took those top-50 predicted associations verified by three benchmark databases (i.e. HMDD v2.0, dbDEMC and miR2Disease) into consideration. Namely, those predicted associations additionally verified by extra literatures were not included. Besides, we could only compare performance between PBMDA and other compared models for those given diseases, whose verification was published in their articles, e.g. prostatic, breast and lung neoplasms in HDMP. Based on these comparison results (see Table 10), our model generally improves prediction performance for various selected diseases, relative to other compared computational models.

**Table 10:** We implemented PBMDA model on the older version of HMDD (v1.0) for further comparison between PBMDA and another three representative computational models, i.e. HDMP, RWRMDA and RLSMDA. For these given diseases, their top-50 prediction lists have been verified by three benchmark databases (i.e. HMDD v2.0, dbDEMC and miR2Disease).

	Prostatic neoplasms	Breast neoplasms	Lung neoplasms
PBMDA	44	48	41
HDMP	38	43	38
	Breast neoplasms	Colon neoplasms	Lung neoplasms
PBMDA	48	38	41
RWRMDA	48	33	43
	Hepatocellular cancer	Colon cancer	
PBMDA	38	38	
RLSMDA	40	36	

To prove the applicability of our model to a new disease (no known associated miRNAs), we selected *Glioblastoma* for further verification. We removed all records about *Glioblastoma* from the known miRNA-disease association network derived from HMDD and that *Glioblastoma* could be regarded as a new disease. We implemented our model for prediction and then the prediction result about *Glioblastoma* was yielded. Similarly, we verified top-50 predicted miRNA-disease associations by HMDD, dbDEMC and miR2Disease (see Table 11). Fifteen out of top-20 and 37 out of top-50 predicted miRNAs have been verified to be associated with *Glioblastoma* by HMDD, dbDEMC and miR2Disease. Based on these prediction results, we can safely conclude that PBMDA can still achieve the reliable prediction performance for a new disease. Most importantly, it also demonstrates that our model is indeed applicable to a new disease.

**Table 11:** We removed all records about *Glioblastoma* from the known miRNA-disease association network derived from HMDD and implemented our model for prediction. Fifteen out of top-20 and 37 out of top-50 predicted miRNAs have been verified to be associated with *Glioblastoma* by HMDD, miR2Disease and dbDEMC databases.

Top 1–25		Top 26–50	
miRNA	Evidence	miRNA	Evidence
hsa-mir-21	HMDD;miR2Disease;dbDEMC	hsa-mir-574	unconfirmed
hsa-mir-155	HMDD;dbDEMC	hsa-mir-208a	HMDD
hsa-mir-222	HMDD;miR2Disease;dbDEMC	hsa-mir-181a	HMDD;miR2Disease;dbDEMC
hsa-mir-221	HMDD;miR2Disease;dbDEMC	hsa-mir-181c	HMDD;miR2Disease;dbDEMC
hsa-mir-122	unconfirmed	hsa-mir-132	dbDEMC
hsa-mir-199a	dbDEMC	hsa-mir-214	dbDEMC
hsa-mir-205	HMDD;dbDEMC	hsa-mir-125b	HMDD;miR2Disease
hsa-mir-451a	HMDD	hsa-mir-25	HMDD;miR2Disease;dbDEMC
hsa-mir-93	unconfirmed	hsa-mir-182	dbDEMC
hsa-mir-451	miR2Disease	hsa-mir-181b	HMDD;miR2Disease;dbDEMC
hsa-mir-142	HMDD;dbDEMC	hsa-mir-1	unconfirmed
hsa-mir-206	HMDD;dbDEMC	hsa-mir-9	HMDD;miR2Disease;dbDEMC
hsa-mir-34a	HMDD;miR2Disease;dbDEMC	hsa-mir-641	unconfirmed
hsa-mir-27a	unconfirmed	hsa-mir-1287	unconfirmed
hsa-mir-210	HMDD;dbDEMC	hsa-mir-1286	unconfirmed
hsa-mir-10b	HMDD;miR2Disease;dbDEMC	hsa-mir-22	HMDD
hsa-mir-23b	HMDD;miR2Disease	hsa-mir-1290	unconfirmed
hsa-mir-320c	unconfirmed	hsa-mir-432	dbDEMC
hsa-mir-30b	unconfirmed	hsa-mir-197	miR2Disease;dbDEMC
hsa-mir-27b	HMDD	hsa-mir-19a	HMDD;dbDEMC
hsa-mir-193b	unconfirmed	hsa-mir-34c	dbDEMC
hsa-mir-16	HMDD;dbDEMC	hsa-mir-106b	unconfirmed
hsa-mir-15a	HMDD;dbDEMC	hsa-mir-19b	HMDD;dbDEMC
hsa-mir-17	HMDD;dbDEMC	hsa-mir-18a	HMDD;dbDEMC
hsa-mir-29a	HMDD	hsa-mir-95	HMDD;dbDEMC

As a global computational model, PBMDA was also implemented to simultaneously prioritize the potential miRNAs for all investigated diseases. Owing to the limited prior knowledge, some promising disease-related miRNAs have not been validated yet. We therefore listed the top-100 potential associations in S6 Table.

## Discussions

With the great amount of researches, it was found that miRNAs play increasingly significant roles in many physiological processes including complex human diseases. Researchers attempt to identify disease-related miRNAs as valuable biomarkers for clinical measure, diagnosis, prognosis and treatment. The biological experiment-based verification is not only time-consuming but also expensive, which boosts the development of computational predictive models. A novel Path-Based MiRNA-Disease Association (PBMDA) computational prediction model was proposed here by integrating heterogeneous biological networks. PBMDA could construct a heterogeneous graph by padding internal connections, including miRNA-miRNA similarity, disease-disease similarity and known miRNA-disease associations. MiRNA-miRNA similarity and disease-disease similarity are inferred from Gaussian interaction profile kernel similarity for miRNA and disease, miRNA functional similarity network, and disease semantic similarity network. Compared with four state-of-the-art computational models, PBMDA achieved the highest AUCs of 0.9169, 0.8341 and 0.9172+/-0.0007 in the evaluation frameworks of global LOOCV, local LOOCV and 5-fold CV, respectively, demonstrating the most reliable prediction performance. In the case studies of three important human complex diseases, 44, 44, and 45 of top-50 predicted miRNAs of *Esophageal Neoplasms*, *Kidney Neoplasms* and *Colon Neoplasms* have been experimentally supported by the previous experimental literatures, respectively. By manually validating the predicted obesity-related miRNAs based on the published literatures, 9 out of top-10 predicted miRNAs have been demonstrated to be associated with obesity. Furthermore, through the comparison performance between PBMDA and other previous models in case studies, it is anticipated that PBMDA would significantly accelerate the identification of miRNA-disease associations. We are planning to provide a standalone tool or webserver for users in the future. This study is aimed to firstly propose the computational model for the next schedule.

There are several major factors contributing to the high prediction performance of PBMDA. First, reliable biological datasets were utilized to establish an integrated similarity network, which represents three relationships (i.e., miRNA-miRNA similarity, disease-disease similarity, and miRNA-disease associations). Second, as a path-based model, PBMDA can effectively take advantage of topological information implied in the integrated heterogeneous network. Third, PBMDA can be applied for new disease (no known associated miRNAs) and new miRNAs (no known associated diseases), which greatly improves the practicability and reliability of the PBMDA. Depending on the disease semantic similarity and miRNA functional similarity, we can construct the disease-disease and miRNA-miRNA similarity network. Depth-first search algorithm can be used to assign the scores to the paths like: disease_new_↔disease↔miRNA and miRNA_new_↔miRNA↔disease. In this way, the unverified disease-miRNA associations including new diseases and/or new miRNAs also can be prioritized based on their aggregated scores. Fourth, the model of PBMDA can be easily introduced together with other biological information (e.g. various miRNA-related interactions and disease phenotypic similarity [61,62]) for further improving the quality of the integrated heterogeneous network. Last but not least, PBMDA could simultaneously prioritize candidate miRNAs for all investigated diseases.

There is still a vast potential to boost the prediction performance of PBMDA, which still have some limitations. For examples, the miRNA-disease associations obtained from HMDD database are far from enough, which greatly influences the performance of our approach. The disease semantic similarity and miRNA functional similarity have problem of sparsity, which was remedied by integrating the Gaussian interaction profile kernel similarity inferred from the known miRNA-disease associations. It inevitably did bring the predicted error to the constructed heterogeneous graph. Finally, the distance-decay function in our approach is relatively simple, and it could be reconstructed based on the machine learning methods.

## Supporting information

S1 Table Known human miRNA-disease associations obtained from HMDD database. (XLSX)

S2 Table Names of 495 miRNAs involved in known human miRNA-disease associations obtained from HMDD database. (XLSX)

S3 Table Names of 383 diseases involved in known human miRNA-disease associations obtained from HMDD database. (XLSX)

S4 Table The constructed miRNA functional similarity score matrix. (XLSX)

S5 Table PBMDA’s top-50 prediction list verified by dbDEMC and miR2Disease databases. (XLSX)

S6 Table As a global measure model, PBMDA can simultaneously prioritize all unknown potential related miRNAs for all investigated diseases. We here publicly released the top-100 potential miRNA-disease associations predicted by PBMDA.
(XLSX)
